# Medical Media in Pediatric Gastroenterology and Hepatology: Strategies for Effective Use by Consumers, Contributors, and Creators

**DOI:** 10.1007/s11894-024-00947-3

**Published:** 2024-10-17

**Authors:** Temara Hajjat, Paul Tran

**Affiliations:** 1 Cincinnati Children’s Hospital and Medical Center, University of Cincinnati, Cincinnati, OH USA; 2 Phoenix Children’s Hospital, University of Arizona College of Medicine, Phoenix, AZ USA

**Keywords:** Medical media, Social media, Promotion, Medical education

## Abstract

**Purpose of the Review:**

This review article explores the role of medical media in gastroenterology and hepatology, highlighting its benefits for enhancing clinical practice, education, and patient engagement. It also provides practical guidance for gastroenterologists and hepatologists on effectively implementing these tools in their daily practice.

**Recent Findings:**

Recent findings highlight that medical media significantly boosts citation rates and dissemination of research, enhances promotional efforts, and fosters greater engagement from patients and trainees. These advances underscore the growing role of medical media in amplifying academic impact and improving educational outreach in gastroenterology and hepatology.

**Summary:**

Integrating medical media into pediatric gastroenterology and hepatology offers numerous benefits, from enhanced education and professional development to improved patient engagement. By understanding the roles of contributor, creator, and consumer and leveraging the right platforms and content types, pediatric gastroenterologists and hepatologists can effectively utilize medical media to advance their field and provide better care.

## Introduction

In its various forms, media serves as a powerful tool for communicating and disseminating information across diverse audiences. Traditionally encompassing newspapers, television, radio, and, more recently, digital platforms, media has evolved to become an integral part of daily life, influencing public opinion, culture, and knowledge.

In the healthcare sector, the emergence of medical media—specialized content dedicated to healthcare-related information—has transformed how information about health and medicine is shared and consumed. Medical media, which includes platforms such as medical journals, websites, mobile apps, social media, and podcasts, plays a pivotal role in education for healthcare professionals, patients, and the public. Medical media, particularly for "edutainment"—a blend of education and entertainment—can take various forms, including blogs, podcasts, webinars, and social media posts. These platforms serve as vehicles for professional communication and as tools for patient education and engagement.

The intersection of media and healthcare has given rise to new opportunities and challenges in disseminating medical information. Social media has become an increasingly popular avenue for individuals to seek out healthcare information. A growing number of individuals now turn to social media platforms to seek out healthcare information, driven by the accessibility and immediacy these platforms offer [1]. Moreover, the use of media, particularly digital platforms, has become increasingly common for obtaining healthcare information, reflecting a broader shift in how patients and the public engage with medical content [2].

Healthcare practitioners have also begun to recognize the potential of media platforms as tools for professional engagement. Many clinicians use social media and other digital channels to interact with patients, providing a more direct and personalized approach to patient care [3]. Additionally, these platforms offer opportunities for career advancement, allowing practitioners to build their professional networks, showcase their expertise, and stay informed about the latest developments in their field [4]. Media platforms are further utilized for patient education, enabling healthcare providers to offer accurate and timely information to a broader audience, thus enhancing patient understanding and adherence to medical advice [5]. Furthermore, media serves as a valuable communication tool among healthcare professionals, facilitating collaboration, knowledge sharing, and professional development [6].

However, engaging in medical media can be daunting for some healthcare providers. The fear of public scrutiny, concerns about professionalism, and the potential for misinformation are significant barriers that may prevent clinicians from fully participating in these platforms [7]. These challenges underscore the need for clear guidelines and practical strategies to help healthcare providers navigate the complexities of medical media effectively. Integrating medical media into professional practice may seem daunting at first, but it is more manageable than it appears. One must understand the roles within this landscape: consumers, contributors, and creators. Each role offers unique opportunities and responsibilities in disseminating and consuming medical content.

This review will explore the landscape of medical media, providing a comprehensive overview of its applications and implications for healthcare practitioners, particularly pediatric gastroenterologists and hepatologists. It will focus on the impact and utilization of medical media among pediatric gastroenterologists and hepatologists, exploring how different platforms can be leveraged for professional development and patient education. This review aims to build upon existing literature regarding the application of media in the field of pediatric gastroenterology [8–10]. It will also offer practical tips and strategies for engaging with medical media, helping providers harness these platforms for professional growth, patient education, and effective communication within the healthcare community.

## Social Media vs. Medical Media

In this paper, we propose adopting the term "medical media" when discussing different forms of media utilized for the dissemination of medical knowledge, thereby distinguishing it from the established concept of "social media." By integrating this terminology, we aim to broaden the discussion to include the various media platforms used by pediatric gastroenterologists and hepatologists, thereby enhancing our understanding of how these tools can support clinical practice and educational efforts.

Medial media refers to the specialized use of digital platforms and tools by healthcare professionals to share knowledge, develop professionally, and promote public health education. In pediatric gastroenterology and hepatology, medical media includes digital mediums such as the specialized webinars offered by the North American Society for Pediatric Gastroenterology, Hepatology, and Nutrition (NASPGHAN) and podcasts such as "Bowel Sounds," [11] which provides insights into up-to-date practices and research in pediatric gastroenterology and hepatology. These platforms are curated to meet the specific needs of healthcare professionals, ensuring that content is accurate, evidence-based, and relevant to clinical practice.

On the other hand, social media platforms like X (formaly twitter), Facebook, Instagram, and TikTok, although not initially created for medical use, have become important tools for pediatric gastroenterologists and hepatologists. When these platforms are utilized to share medical information—such as discussing clinical cases, raising awareness about pediatric gastrointestinal conditions, or educating patients and peers—they function as a subset of medical media. For instance, on X (formaly twitter), the hashtag #PedsGI is frequently used by pediatric gastroenterologists to share updates on new research, promote their research, and highlight upcoming events related to pediatric gastroenterology. On TikTok, pediatric gastroenterologists such as “@alimentaryschool” have created short videos explaining symptoms, treatment options, and preventive measures for gastrointestinal disorders in children, reaching a younger and more diverse audience.

Incorporating social media into medical media has transformed how pediatric gastroenterologists and hepatologists communicate and disseminate information. It enables real-time updates, encourages global collaboration, and offers effective public health outreach. However, this integration also requires careful consideration of the platform's limitations, such as the potential for misinformation and the need to maintain professional integrity in a public forum.

## Role of Medical Media in Career Advancements

Medical media platforms have increasingly become crucial for advancing medical careers by promoting academic work, increasing visibility of research, and fostering professional networking [8, 9, 13]. These platforms provide unique and valuable opportunities for physicians and researchers to share their work with a broader audience, including peers, patients, and the public. Evidence suggests that the use of medical media is linked to higher citation rates of academic papers and greater engagement within professional networks [14, 15]. This heightened engagement can lead to significant career advancement opportunities, such as invitations to present at conferences or collaborate on research projects. Moreover, medical media platforms are valuable for medical education [16]. Incorporating medical media into education for pediatric gastroenterology and hepatology trainees enhances knowledge acquisition by providing access to diverse and up-to-date educational materials, including interactive webinars and podcasts. This approach facilitates a more engaging and flexible learning experience and supports the rapid dissemination of current research findings and clinical insights, thereby improving overall educational outcomes and professional development.

Medical media platforms also play a key role in building a professional brand and establishing a reputation as a thought leader in specific areas of medicine [17]. Consequently, effectively leveraging these digital tools has become an essential component of career development in the medical field. Recognizing the growing importance of these platforms, some institutions are now incorporating medical media activity into promotion and tenure evaluations [18, 19]. Additionally, active division accounts can increase national rankings and reputation scores [20].

## The Role of “Edutainment”

One of the compelling aspects of medical media is its potential for "edutainment"—a blend of education and entertainment. This approach makes learning more engaging and accessible, particularly in fields requiring continuous education, like pediatric gastroenterology and hepatology. Edutainment targets medical personnel, learners, and the public. Various types of media can be employed for edutainment, including podcasts, infographics, animated videos, interactive webinars, and even gamified learning experiences, which can be utilized to create edutaining, informative, and enjoyable content, convey important medical information and engage the audience through entertaining content.

## Navigating Medical Media: Roles of Pediatric Gastroenterologists and Hepatologists as Consumers, Contributors, and Creators

Engaging with medical media might initially seem daunting, but it can be approached systematically. Pediatric gastroenterologists and hepatologists can engage with medical media in three main roles: consumer, contributor, and creator. These roles are fluid and can overlap, allowing individuals to transition between them based on their professional needs and personal interests. For clarity, this paper will define each role separately while acknowledging their interconnectedness. Understanding these roles—consumer, contributor, and creator—will help pediatric gastroenterologists and hepatologists strategically leverage medical media to enhance their professional development, contribute to the medical community, and influence public health discourse. Table 1 summarizes the different roles with practical tips and pearls for engaging in each, while Table 2 provides guidance on content creation and platform selection.

**Table 1:** Tips and Pitfalls for Pediatric Gastroenterologists and Hepatologists in Medical Media Roles

Role	Activities	Practical tips	Possible pitfalls
Consumer	Follows and learns from content, engages with resources, reads, watches, or listens to content created by others.	**Curate Credible and Relevant Information:** Follow active researchers, major journals such as Gastroenterology, Current Gastroenterology Reports, JPGN, and social media accounts of relevant societies. **Collect and Share:** Build collections of medical media across different domains for educational purposes (e.g., expert interview podcast episode, YouTube explanation, informational graphic from Instagram on new-generation biologic medications for inflammatory bowel disease.	**Overconsumption:** Unchecked social media consumption can lead to inefficient use of time. Consider discrete blocks of time within a schedule to use medical media. **Workplace Considerations:** Some workplaces may limit or fully restrict access to medical media. Consider having backups or accessible versions on local servers when using them for educational purposes, such as presentations.
Contributor	Shares existing content and/or comment and engage in professional discussions.	**Engage People, Not Just Content:** Actively participate in online discussions, offer commentary, and engage with peers and audiences to foster a dynamic exchange of ideas. **Promote Information:** Utilize social media to highlight and promote professional and academic work, upcoming conferences, or new publications, thereby amplifying the reach of critical medical information. Patients, learners, or other healthcare professionals may appreciate direction to new research. **Combat Misinformation: C**ombating misinformation by sharing accurate, evidence-based information.	**Feeding Trolls:** Avoid abusive or heated exchanges, with online commenters who may be trying to provoke a reaction. Consider professional language in all online posts, forums, and comments. **Burnout:** Working to combat misinformation can be emotionally exhausting. Consider engaging with other contributors or creators to “share the load”.
Creator	Produces original medical content such as articles, videos, podcasts, blogs, etc.	**Choose Your Content Wisely:** Identify topics you are passionate about and have expertise in, considering what makes your perspective unique and valuable to your intended audience. Find your niche and provide unique insights or perspectives that are not widely covered. Focus on areas where you have the most knowledge and can provide the most value. Establishing credibility is key to building an audience. Develop content that reflects your true passion. This may naturally contain more energy or enthusiasm, and your audience may find content that is true to your purpose more resonant. **Identify your Target Audience:** Define your targeted consumers and determine what content is the most resonant or valuable to them. For instance, learners may appreciate advice for professional development, whereas patients will appreciate approachable explanations of common conditions. **Plan for Logistics:** Consider the technical aspects of content creation, such as equipment needs, editing skills, and the time investment required. Batch-produce and repurpose content that can be disseminated on a regular schedule across a variety of domains and platforms. **Cultivate Community Engagement:** Effective content creation involves producing material and engaging with your audience through comments, response videos, and live events to build and sustain a community around your content. **Intellectual Property and Professionalism:** Maintain professionalism and protect your intellectual property. Ensure that your content reflects your professional values, safeguards intellectual property rights, and builds your personal brand in a way that supports your career goals.	**Intellectual Property and Copyright:** Your employee agreement may include stipulations on ownership of any medical media produced or requirements for disclaimers. Consider reviewing your employer’s social media and intellectual property policies. **Directionless Content:** Produced medical media may be too general, lack a clear purpose, or bounce between too many domains. Consider a clear goal for why content should be produced and who the target audience should be. **Offering Medical Advice:** Educational content may be misconstrued as medical advice, and care must be taken to present information solely for educational or entertainment purposes. Consider including a statement such as: “Information presented is intended for education or entertainment, expresses opinions, and should not be taken as medical advice.”

**Table 2:** Content Creation and Platform Selection

Platform	Suitable content types	Examples in pediatric gastroenterology	Practical tips	Possible pitfalls
YouTube	Educational videos, webinars, patient education videos	Video Journal and Encyclopedia of GI Endoscopy https://www.youtube.com/@videojournalandencyclopedi3980 “Olympus Colonoscopy Training Videos” https://www.youtube.com/playlist?list=PLM0XNaMipf51ASoOWTUGDXoxRJGSlYILn	Storyboard prior to recording by writing word-for-word script with representative images or scenes for a smoother production process and more effective visual aids Use a conversational tone and informal communication style to increase engagement and attention Add one interactive element every 5–10 s to improve retention Have a reviewer watch each video before sharing	Videos with poor audio or video quality increase a viewer’s cognitive load and can decrease effectiveness or retention Dull or undescriptive thumbnails may limit the audience who clicks on the video
Podcast	Interviews, case discussions, expert insights	Bowel Sounds: The Pediatric GI Podcast https://naspghan.buzzsprout.com/	Incorporate dialogue with a conversational tone, personal anecdotes, and humor to create a more engaging experience For educational podcasts, include credible source material and summarize key points Consider adding the ability to navigate quickly to the desired content	Users may not know of the podcast. Therefore promotion across other social media venues may help organic word-of-mouth discovery Unengaging podcasts can lead to a lack of retention and prevent users from subscribing Lack of scheduled content can lead to loss of subscribers
TikTok	Short educational clips, quick tips, patient interaction	https://www.tiktok.com/@kidsgastrodoc	Emphasize an audio, video, and/or text “hook” in the first 3 s of a video to capture a viewer’s attention and varied pace to keep them engaged Use on-screen text, titles, captions, and hashtags with similar words for search engine optimization (SEO) Creating a series of videos with similar design templates or design concepts can streamline production and editing	A prolonged introduction or pleasantries at the beginning of a video may quickly disengage a viewer Failure to engage with viewer comments and questions can limit profile growth “Talking head” style videos can risk being meandering and boring to viewers. Consider the use of storytelling and pop-ups with text or images to engage the viewer
Blog	In-depth article reviews, patient stories, research summaries, opinions and experiences	Guts and Growth https://gutsandgrowth.com/	Use hyperlinks to relevant websites or other posts on the blog to refer readers to related content Use images and pictures to break up text	Promotion committees may view blog entries less favorably than traditional publication activities Keep scientific rigor and meaningful contributions for work to be valued
X (formally known as “Twitter”)	Promotion of new research and networking opportunities, educational tips and diagrams	https://x.com/MondayNightIBD https://x.com/NASPGHAN https://x.com/AustinChiangMD	Create thoughtful and easy-to-follow “tweetorials” or mini-lectures with high-yield resources for learners Engage with like-minded users to foster a sense of community	Strong or emotional language may be regarded as inflammatory or reflect poorly on employers Prior to posting, consider looking at a draft on a non-posting application such as Notes or texting to someone else to review
Instagram	Personal and professional updates, pictures, and short-form video content	https://www.instagram.com/alimentaryschool	Utilize Instagram stories to show more “in-the-moment” or spontaneous insights into the day-to-day moments that can help viewers feel connected to a creator A mix of image and video content may be engaging to viewers with different consumer preferences Mirroring content on other platforms, such as X or TikTok, can expand reach to other viewers	A block of text over a picture may lead to less audience engagement Repurposed content (especially from TikTok) can include another platform’s watermark, which can lead to less promotion on Instagram Reels. Consider using native video without a watermark

### The Consumers of Medical Media: Passive Engagement for Knowledge and Networking

In the realm of pediatric gastroenterology and hepatology, consumers of medical media are professionals who utilize these platforms for various purposes, such as professional development, personal “edutainment,” staying updated on current literature and conferences, and networking with colleagues [21]. These individuals primarily engage with content created by others, focusing on absorbing information rather than actively producing or disseminating it themselves. While consumers may occasionally share content, their main objective is not to educate a broader audience but to meet their own informational needs.

Pediatric gastroenterologists and hepatologists often act as consumers when viewing social media posts about recent conferences, listening to podcasts, attending webinars, or networking with colleagues through online platforms. This passive engagement is common among healthcare practitioners, including those in pediatric subspecialties [22]. Despite the passive nature of their interaction, consumers can still influence the field; for instance, sharing or “retweeting” a journal post can significantly increase an article's visibility and citations [14, 20]. Therefore, even in a consumer role, individuals contribute to the broader dissemination of information.

For those in the consumer role, it is essential to identify and engage with platforms tailored to their specific professional interests to maximize the benefits of their media consumption. By thoughtfully selecting relevant content, consumers enhance their knowledge and professional growth and support content creators by engaging with and promoting the creator’s work within the medical community.

Consumers of medical media face several pitfalls, including excessive time spent on social media platforms, which can lead to information overload and decreased productivity [23]. Overreliance on social media can also expose individuals to unverified or misleading information, contributing to confusion or misinformation. Additionally, frequent engagement with social media can reduced time for active learning and professional development.

To combat these issues, it is essential for consumers to set clear boundaries on their social media usage and prioritize time management strategies by incorporating scheduled breaks, establishing structured routines for engaging with medical media, and setting specific time limits to avoid excessive use. Prioritizing reputable sources, engaging with high-quality peer-reviewed sources, and using curated platforms dedicated to professional development can help ensure that the information consumed is accurate and relevant. Furthermore, diversifying media consumption—such as subscribing to reputable journals, listening to podcasts, and attending accredited webinars— and leveraging tools and apps designed to filter high-quality content can mitigate the risk of misinformation and enhance the overall effectiveness of medical media engagement.

### The Contributors of Medical Media: Active Participation by Enhancing Interaction and Dissemination of Medical Knowledge

Contributors play a pivotal role in disseminating and discussing medical media content by actively engaging with and sharing information. Unlike consumers who passively absorb content, contributors participate in discussions by commenting on posts, resharing content, and providing information from conferences. While they do not focus on creating original content, contributors significantly enhance the visibility of conferences, research findings, and new publications within their field. Their involvement is crucial in addressing the challenge of online health misinformation by sharing accurate, evidence-based information, thus helping to ensure that patients and the public receive reliable guidance.

Contributors also facilitate health-related research by helping recruit diverse participant cohorts for collaborative studies [24]. Platforms like X (Formally Twitter), YouTube, Facebook, Instagram, and TikTok enable contributors to comment on original content and engage in community discussions, which can help enhance and reach medical media overall [25]. It is in comments that contributors can establish credibility themselves, improving the quality of discourse by addressing misinformation in topics such as neuropsychiatric side effects in children with the use of PEG 3350.

Contributors to medical media face several pitfalls, including the potential for engaging with trolls, spreading misinformation, and encountering challenges related to content quality and accuracy. Engaging with trolls—individuals who post inflammatory or disruptive comments—can detract from productive discussions and create a hostile environment. This interaction often shifts focus away from substantive content and can undermine the credibility of the contributor and the overall discourse. To address this issue, contributors should maintain a professional demeanor, avoid responding to provocations, and focus on promoting accurate information. Utilizing platform moderation tools and reporting mechanisms can help manage and minimize interactions with disruptive users.

Another significant challenge is the risk of spreading misinformation. Contributors might unintentionally share incorrect or outdated information, perpetuating misconceptions and potentially harming public health [26–28]. To mitigate this risk, contributors should ensure that the content they share is evidence-based and derived from reputable sources. Cross-referencing information with current research and guidelines can help verify its accuracy. Additionally, contributors should be transparent about their information sources and provide references to support their claims.

Moreover, contributors may face difficulties in managing the quality of their content and ensuring it remains relevant and accurate. Regular updates and peer reviews of shared content can help maintain its credibility and relevance. Contributors should engage in continuous education and stay informed about the latest developments in their field to provide high-quality and accurate information.

Overall, addressing these pitfalls involves a combination of maintaining professionalism, ensuring the accuracy of information, and using available tools to manage interactions and content effectively.

### The Creators of Medial Media: Generating Original Content Creation by Innovating and Leading in Medical Media

Creators in medical media play a highly proactive role by producing original content designed for specific audiences with the intent of broad dissemination and engagement. For pediatric gastroenterologists and hepatologists, this includes creating diverse media formats such as social media posts, blogs, videos, and podcasts aimed at education, edutainment, and professional interaction. The process of becoming a creator involves strategic planning, which includes aligning content with one’s expertise and interests, selecting appropriate platforms for distribution, addressing intellectual property and copyright issues, and upholding professional standards. These content creators act as educators, utilizing various medical media channels to foster microlearning environments that enhance learner confidence, support professionalism, and encourage collaborative learning [29, 30].

Content creation also provides opportunities to influence public discourse, share specialized knowledge, and develop a professional brand. Pediatric gastroenterologists and hepatologists should follow several key steps to achieve success as creators, which are detailed below.

## Content Creation and Platform Selection

The initial step in creating medical media content involves developing material that aligns with one's expertise and passion while ensuring authenticity and authority. This process requires understanding the target audience and producing educational content that is both informative and engaging. Content creation can extend beyond clinical applications and patient care. For instance, “The White Coat Investor,” a widely recognized platform created by an emergency room physician, offers educational content on financial literacy specifically for physicians. This platform serves as a resource for financial literacy, personal finance, and investing tailored to medical professionals [31].

Selecting the appropriate media format and platform is crucial to enhance reach and impact when creating medical content. Different platforms cater to various audience demographics and content types, each with its own benefits and limitations. For instance, YouTube is well-suited for longer educational videos, such as tutorials and animated demonstrations of endoscopic procedures. An example is the Olympus Corporation’s video series, which provides comprehensive tutorials for novice endoscopists learning colonoscopy techniques [32]. In contrast, TikTok and Instagram Reels are effective for short, engaging videos but may lack depth. The account “@alimentaryschool” exemplifies this by offering brief educational clips on gastroenterology topics like eosinophilic esophagitis and constipation on both TikTok and Instagram [12]. The platform X is useful for promoting new research and sharing educational mini lectures, known as "tweetorials," while LinkedIn primarily serves as a networking and career development tool [21].

To distinguish themselves amidst the extensive content available, creators must enhance engagement by providing unique perspectives or specialized expertise. For longer YouTube videos, effective strategies include storyboarding and scripting, which can streamline production and improve visual aids. Incorporating a conversational tone, informal dialogue, and interactive elements can boost viewer engagement and attention [33]. Adding "chapters" to YouTube videos enables viewers to access specific information directly without watching the entire video. For Instagram and TikTok, employing clear titles, eye-catching thumbnails, and presenting high-yield material within the first 3 seconds can enhance viewer retention. Utilizing on-screen text, captions, and relevant hashtags can improve search engine optimization (SEO) [34]. Podcasts benefit from a conversational tone, personal anecdotes, and humor. Educational podcasts should include credible sources and summaries of key points [33]. On X, "tweetorials" or “Twitter chats” effectively break down complex information and curate valuable resources for learners [8].

## Community Engagement and Combating “Trolls”

Community engagement significantly impacts the growth of media creators. Active engagement strategies can foster more enthusiastic contributors, such as responding to comments, conducting question-and-answer sessions, providing audio or video responses, and incorporating consumer feedback into new media. This helps build a more engaged community around the content.

Effective promotion and engagement are vital for reaching a broader audience. Key strategies include cross-platform sharing, maintaining consistent posting schedules, and creating interactive content [34, 35]. Promoting content involves implementing SEO strategies, utilizing social media platforms, and regularly interacting with followers. Optimizing titles, captions, and hashtags enhances content discoverability and extends its reach [34].

Creators of medical media can employ several strategies to combat trolls and maintain a constructive environment. Ignoring trolls is effective. Engaging with them can escalate conflicts and draw further attention to their disruptive behavior. Instead, creators should focus on fostering positive interactions with their audience. Reporting abusive or inappropriate content to platform administrators can help address issues at a systemic level, ensuring that such behavior is mitigated. Additionally, implementing moderation tools and setting clear community guidelines can prevent trolls from gaining traction. Creators should also consider using pre-emptive measures, such as disabling comments on specific content or filtering certain keywords, to reduce the likelihood of encountering negative interactions. Engaging with professional organizations or platforms that offer resources on managing online abuse can further support creators in maintaining a safe and respectful online presence. These strategies collectively contribute to a more productive and secure environment for both content creators and their audiences.

## Technical Considerations

A smartphone provides content creators with access to a wide array of apps for developing and distributing medical media. For instance, while TikTok is primarily recognized as a short-form video platform, it also offers robust editing tools that enable users to film, edit, and post videos and photos [25]. User-friendly applications like “Canva”, which offers pre-made templates for thumbnails and image posts, simplify the content creation process for beginners, eliminating the need for extensive equipment or advanced editing skills. Furthermore, repurposing content across different platforms, such as using audio from a YouTube video for a podcast or segmenting the video for posts on TikTok, Instagram, or Facebook, can streamline content creation and reduce the time required for producing new material. Scheduling content releases in advance can also alleviate the burden of timely posting.

Effective content creation involves acquiring the right equipment, editing content, and managing schedules. Building a solid online presence through audience engagement—via comments, Instagram/TikTok stories, and live interactions—along with cross-platform promotion, enhances engagement, fosters peer collaboration, and cultivates an interactive community [34, 35].

## Professionalism and Intellectual Property

Consideration of intellectual property (IP), monetization options, and copyright is essential for maintaining professionalism and protecting and potentially profiting from content. A creator's products inherently reflect their professional image, and it is important to recognize that content may reach or be engaged with by individuals outside the intended audience. Creators should be mindful of potential conflicts between their personal views and those of their employer. Ensuring that content aligns with the values and legal standards of the employer is crucial for upholding professionalism [21].

Content created may be considered intellectual property owned by the employer. It is advisable to review contracts or employee agreements related to medical media and intellectual property prior to content development [36]. Consideration of waiving the employer’s rights to intellectual property may also be relevant, but understanding the associated terms is essential.

By addressing these factors, pediatric gastroenterologists and hepatologists can establish themselves as thought leaders, make meaningful contributions to their field, and advance their careers through medical media. Developing a distinctive personal brand can enhance visibility and influence within the medical community, significantly benefiting the field of pediatric gastroenterology and hepatology.

## Conclusion

Integrating medical media into pediatric gastroenterology and hepatology presents significant advantages for advancing both the field and the quality of patient care. Medical media platforms serve as vital tools for gastroenterologists and hepatologists by enhancing medical education, facilitating access to diverse educational resources, and promoting continuous professional development. By understanding and embracing the roles of contributor, creator, and consumer within these platforms, pediatric gastroenterologists and hepatologists can effectively disseminate knowledge, engage with peers, and stay updated with the latest research and clinical practices.

Moreover, medical media plays a crucial role in combating misinformation and addressing gaps in information dissemination. By leveraging credible sources and high-quality content, gastroenterologists and hepatologists can ensure that their audience receives accurate and evidence-based information, thereby avoiding potential pitfalls associated with unreliable or outdated content.

This paper has provided practical tools and strategies for gastroenterologists and hepatologists interested in incorporating medical media into their professional practice. By utilizing these tools and understanding the intricacies of various platforms, practitioners can enhance their visibility, foster professional networks, and ultimately contribute to the advancement of pediatric gastroenterology and hepatology. Integrating medical media thoughtfully into their practices can lead to better educational outcomes and improved patient care.

## Key References

- Chan TM, Dzara K, Dimeo SP, Bhalerao A, Maggio LA. Social media in knowledge translation and education for physicians and trainees: a scoping review. Perspect Med Educ. 2022;9: 20‐30 doi:10.1007/s40037-019-00542-7
  o This article examines the role of social media in shaping professional identity and its integration into medical education, emphasizing its impact on learning and career development.
- Silverman JA, Chugh A, Hollier JM, et al. Using social media for patient care, research, and professional development: a North American Society of Pediatric Gastroenterology, Hepatology, and Nutrition position paper. *J Pediatr Gastroenterol Nutr*. 2024;78(2):414–427 doi:10.1002/jpn3.12051.
  o This position paper outlines how social media can be utilized for patient care, research, and professional development within pediatric gastroenterology, providing guidelines and recommendations.
- Chiang AL, Rabinowitz LG, Alakbarli J, Chan WW. The Patterns and Impact of Social Media Exposure of Journal Publications in Gastroenterology: Retrospective Cohort Study. J Med Internet Res 2021;23(5):e25252 doi: 10.2196/25252 PMID: 33707166PMCID: 8,164,116
  o This research investigates the independent association between social media exposure and increased citations of gastroenterology publications, emphasizing the role of digital engagement in scholarly impact.
- Sotto-Santiago S, Sharp S, Mac J. The power of social media in the promotion and tenure of clinician educators. *Med Educ Online*. 2023;28(1):10,943 doi: 10.15766/mep_2374-8265.10943.
  o This article discusses how social media can influence the promotion and tenure process for clinician educators, offering insights into its role in academic advancement.

## Data Availability

No datasets were generated or analysed during the current study.

## References

[1] Hesse BW, Nelson DE, Kreps GL, et al. Trust and sources of health information: the impact of the internet and its implications for health care providers: findings from the first Health Information National Trends Survey. Arch Intern Med. 2005;165(22):2618–24. 10.1001/archinte.165.22.2618. 16344419 10.1001/archinte.165.22.2618

[2] Fox S, Duggan M. Health Online 2013. Pew Research Center; 2013.

[3] Forgie EME, Lai H, Cao B, Stroulia E, Greenshaw AJ, Goez H. Social media and the transformation of the physician-patient relationship: viewpoint. J Med Internet Res. 2020;22(6) 10.2196/18165. 10.2196/25230 PMC8742211 34951596

[4] Chan TM, Dzara K, Dimeo SP, Bhalerao A, Maggio LA. Social media in knowledge translation and education for physicians and trainees: a scoping review. Perspect Med Educ. 2022;9:20–30. 10.1007/s40037-019-00542-7. 10.1007/s40037-019-00542-7 PMC7012997 31834598

[5] Moorhead SA, Hazlett DE, Harrison L, Carroll JK, Irwin A, Hoving C. A new dimension of health care: systematic review of the uses, benefits, and limitations of social media for health communication. J Med Internet Res. 2013;15(4)e1933. 10.2196/jmir.1933 PMC3636326 23615206

[6] Bosslet GT, Torke AM, Hickman SE, Terry CL, Helft PR. The patient-doctor relationship and online social networks: results of a national survey. J Gen Intern Med. 2011;26(10):1168–74. 21706268 10.1007/s11606-011-1761-2 PMC3181288

[7] Grajales FJ, Sheps S, Ho K, Novak-Lauscher H, Eysenbach G. Social media: A review and tutorial of applications in medicine and health care. J Med Internet Res. 2014;16(2):e2912. 10.2196/jmir.2912 PMC3936280 24518354

[8] Silverman JA, Chugh A, Hollier JM, et al. Using social media for patient care, research, and professional development: a North American Society of Pediatric Gastroenterology, Hepatology, and Nutrition position paper. J Pediatr Gastroenterol Nutr. 2024;78(2):414–27. 10.1002/jpn3.12051. 38299267 10.1002/jpn3.12051

[9] Bilal M, Oxentenko AS. The impact of Twitter: why should you get involved, and tips and tricks to get started. Am J Gastroenterol. 2020;115(10):1549–52. 32740087 10.14309/ajg.0000000000000763

[10] Chiang AL, Vartabedian B, Spiegel B. Harnessing the hashtag: a standard approach to GI dialogue on social media. Am J Gastroenterol. 2016;111:1082–4. 10.1038/ajg.2016.259. 27349338 10.1038/ajg.2016.259

[11] Bowel Sounds: The Pediatric GI Podcast. Available at: https://naspghan.buzzsprout.com/. Accessed August 15, 2024.

[12] TikTok. Available at: https://www.tiktok.com/@alimentaryschool.

[13] Bardus M, El Rassi R, Chahrour M, et al. The use of social media to increase the impact of health research: systematic review. J Med Internet Res. 2020;22(6). 10.2196/15607 10.2196/15607 PMC7380994 32628113

[14] Smith ZL, Chiang AL, Bowman D, Wallace MB. Longitudinal relationship between social media activity and article citations in the journal gastrointestinal endoscopy. Gastrointest Endosc. 2019;90:77–83. 10.1016/j.gie.2019.03.028. 30935934 10.1016/j.gie.2019.03.028

[15] Chiang AL, Rabinowitz LG, Alakbarli J. Chan WW The Patterns and Impact of Social Media Exposure of Journal Publications in Gastroenterology: Retrospective Cohort Study. J Med Internet Res. 2021;23(5): e25252. 10.2196/25252PMID:33707166PMCID:8164116. 33707166 10.2196/25252 PMC8164116

[16] Yardley S, Dornan T. Kirkpatrick’s levels and education ‘evidence.’ Med Educ. 2012;46(1):97–106. 10.1111/j.1365-2923.2011.04076.x. 22150201 10.1111/j.1365-2923.2011.04076.x

[17] Guze PA. Using technology to meet the challenges of medical education. Trans Am Clin Climatol Assoc. 2015;126:260–70. 26330687 PMC4530721

[18] Sotto-Santiago S, Sharp S, Mac J. The power of social media in the promotion and tenure of clinician educators. Med Educ Online. 2023;28(1):10943. 10.15766/mep_2374-8265.10943. 10.15766/mep_2374-8265.10943 PMC7431188 32821808

[19] Acquaviva KD, Mugele J, Abadilla N, et al. Documenting social media engagement as scholarship: a new model for assessing academic accomplishment for the health professions. J Med Internet Res. 2020;22(7). 10.2196/25070 achievements in health professions. 10.2196/25070 PMC7744266 33263554

[20] Chiang AL, Rabinowitz LG, Kumar A, Chan WW. Association Between Institutional Social Media Involvement and Gastroenterology Divisional Rankings: Cohort Study. J Med Internet Res. 2019;21(9): e13345. 10.2196/13345. 31493321 10.2196/13345 PMC6754684

[21] Chen J, Wang Y. Social media use for health purposes: systematic review. J Med Internet Res. 2021;23(5). 10.2196/17917. 10.2196/17917 PMC8156131 33978589

[22] Hameed I, Oakley CT, Ahmed A, et al. Analysis of Physician Use of Social Media. JAMA Netw Open. 2021;4(7): e2118213. 10.1001/jamanetworkopen.2021.18213. 34292338 10.1001/jamanetworkopen.2021.18213 PMC8299311

[23] Primack BA, Shensa A, Sidani JE, et al. Social media use and perceived social isolation among young adults in the US. Am J Prev Med. 2017;53(1):1–8. 10.1016/j.amepre.2017.01.010. 28279545 10.1016/j.amepre.2017.01.010 PMC5722463

[24] Chang JW, Dellon ES. Challenges and Opportunities in Social Media Research in Gastroenterology. Dig Dis Sci. 2021;66:2194–9. 10.1007/s10620-021-07058-3. 34052939 10.1007/s10620-021-07058-3 PMC8164479

[25] Comp G, Dyer S, Gottlieb M. Is TikTok The Next Social Media Frontier for Medicine?. AEM Educ Train. 2020;5(3):10.1002/aet2.10532 10.1002/aet2.10532 PMC8155692 34095694

[26] Chou W-S, Gaysynsky A, Cappella JN. Where we go from here: Health misinformation on social media. Am J Public Health. 2020;110(Suppl 3). 10.2105/AJPH.2020.305905 10.2105/AJPH.2020.305905 PMC7532328 33001722

[27] George DR, Rovniak LS, Kraschnewski JL. Dangers and opportunities for social media in medicine. Clin Obstet Gynecol. 2013;56(3):500–7. 10.1097/GRF.0b013e318297dc38.PMCID:PMC3863578. 10.1097/GRF.0b013e318297dc38 PMC3863578 23903375

[28] Sheng AY, Gottlieb M, Bautista JR, Trueger NS, Westafer LM, Gisondi MA. The role of graduate medical education in the fight against health misinformation. J Grad Med Educ. 2023;15(1):9–14. 10.4300/JGME-D-22-00383.1. 36817520 10.4300/JGME-D-22-00383.1 PMC9934835

[29] De Gagne JC, Park HK, Hall K, Woodward A, Yamane S, Kim SS. Microlearning in health professions education: scoping review. JMIR Med Educ. 2019;5(2) 10.2196/13997. 10.2196/13997 PMC6683654 31339105

[30] Conde-Caballero D, Castillo-Sarmiento CA, Ballesteros-Yánez I, Rivero-Jiménez B, Mariano-Juárez L. Microlearning through TikTok in higher education: An evaluation of uses and potentials. Educ Inf Technol (Dordr). 2023;2:1–21. 10.1007/s10639-023-11904-4. 10.1007/s10639-023-11904-4 PMC10235824 37361804

[31] The White Coat Investor. Available at: https://www.whitecoatinvestor.com. Accessed August 15, 2024.

[32] Basic Techniques of Colonoscope insertion. Available at: https://www.youtube.com/playlist?list=PLM0XNaMipf51ASoOWTUGDXoxRJGSlYILn

[33] Krumm IR, Miles MC, Clay A, Carlos Ii WG, Adamson R. Making Effective Educational Videos for Clinical Teaching. Chest. 2022;161(3):764–72. 10.1016/j.chest.2021.09.015. 34587482 10.1016/j.chest.2021.09.015 PMC8941608

[34] Lacey H, Price JM. #MedEd—The 'TikTok' frontier of medical education. Clin Teach. 2023 20(5). 10.1111/tct.13636 10.1111/tct.13636 37632321

[35] Plack, D. L., Sharpe, E. E., Wanderman, R. L., Ripoll, J. G., & Abcejo, A. S. (2021). Getting the First Thousand-Optimizing Instagram Residency Content to Increase Followers During the COVID-19 Pandemic. The journal of education in perioperative medicine : JEPM, 23(2), E660. 10.46374/volxxiii_issue2_abcejo 10.46374/volxxiii_issue2_abcejo PMC8168569 34104674

[36] Bloom PP, Matherly S, Kumar S, Koh C. Professional use of social media by hepatology providers. Hepatology. 2021;73(6):2089–91. 10.1002/hep.31824. 33743540 10.1002/hep.31824

